# Perianal Diseases in Pregnancy and After Childbirth: Frequency, Risk Factors, Impact on Women's Quality of Life and Treatment Methods

**DOI:** 10.3389/fsurg.2022.788823

**Published:** 2022-02-18

**Authors:** Diana Bužinskienė, Živilė Sabonytė-Balšaitienė, Tomas Poškus

**Affiliations:** ^1^Clinic of Obstetrics and Gynecology, Faculty of Medicine, Vilnius University, Vilnius, Lithuania; ^2^Clinic of Gastroenterology, Nephrourology, and Surgery, Faculty of Medicine, Institute of Clinical Medicine, Vilnius University, Vilnius, Lithuania

**Keywords:** hemorrhoids, pregnancy, delivery, perianal disease, obstetric

## Abstract

Hemorrhoids and anal fissures occur in about 40% of pregnant women and women during postpartum period. Usually they occur during the third trimester of pregnancy and 1–2 days after giving birth. Constipation during pregnancy, perianal diseases during previous pregnancy and childbirth, instrumental delivery, straining duration of more than 20 min, and weight of the newborn more than 3,800 g are associated with hemorrhoids. Perianal diseases reduce the quality of life of both pregnant and postpartum women. In the absence of acute conditions, surgical treatment of hemorrhoids is delayed after pregnancy, childbirth, and lactation. Thrombosed internal hemorrhoids and perianal thrombosis are to be treated conservatively in most instances by prescribing adequate pain relief, oral, and topical flavonoid preparations.

## Introduction

Pregnancy is a physiological condition; however, pregnant women experience severe anthropometric, physical, metabolic, and psychological changes as well as changes of internal and external organs. These changes can reactivate chronic diseases, present before pregnancy as well as cause new ones (1).

Normal components of the human anal canal are anal cushions (2). They consist of a thickened submucosa, blood vessels, smooth muscle fibers, and connective tissue above the dentate line (2–5). Hemorrhoids is a disease that manifests with symptoms of bleeding from the cushions, their prolapse or vascular space thrombosis (6–8).

Hemorrhoids are classified into external and internal (9–12). External hemorrhoids are vascular spaces below the dentate line, covered by anoderm (3–11). Enlargement and/or clinical symptoms occurring in anal cushions above dentate line are called internal hemorrhoids. They are covered by columnar epithelium and are weakly innervated (3, 4, 10, 11). Internal hemorrhoids are usually painless, even if they prolapse or bleed (9). Only strangulated and thrombosed internal hemorrhoids are very painful. External hemorrhoids are sensitive to palpation (11). Often both external and internal hemorrhoids occur together (10, 13).

It is necessary to distinguish grade IV hemorrhoids—elective, painless situation from the internal thrombosed hemorrhoids—urgent clinical condition accompanied by intense pain, when nodules of internal hemorrhoids suddenly get stuck in the anal canal, thrombosis of the blood vessels occurs and hemorrhoids cannot be pushed back into the anal canal for several days.

## Etiology, Pathogenesis, and Risk Factors

The mechanism of development of hemorrhoids is not entirely clear, but several factors causing this disease have been identified.

During pregnancy, certain mechanical factors increase the development of hemorrhoids.

The growing of the uterus during pregnancy results in increased abdominal pressure in addition to mechanical pressure to the upper part of rectum, inferior vena cava, and portal vein which leads to development of venous stasis, especially in the second part of the pregnancy (4, 14–17). As a result, blood circulation to the internal anal sphincter decreases (4). In addition, during pregnancy, the circulating blood volume increases by 25–40% (3, 12, 15). These factors lead to vascular dilation and venous stasis in pelvis.

Hormonal factors also play an important role in the development of hemorrhoids. The increase in the progesterone can also contribute to the development of hemorrhoids, as it relaxes the walls of your veins, making them more prone to swelling (3–5).

The most common and already proven risk factors are constipation, diarrhea, pregnancy, and childbirth. Pregnancy, childbirth, and the period after childbirth definitely increase the risk of hemorrhoids (4, 5, 18). Natural childbirth is a risk factor for pelvic floor dysfunction (19). Constipation (due to low fluid intake and insufficient amount of fiber in the diet), difficult defecation, venous stasis due to increased abdominal pressure (with increasing uterus), increased volume of circulating blood, hormonal factors (progesterone), obesity, and sedentary lifestyle contribute to the development of hemorrhoids during pregnancy (3–5, 14, 15, 18, 20–23).

Symptoms of anal pathology most commonly occur in the second and third trimesters of pregnancy and after the childbirth (4, 8, 12, 24–27). Risk of developing hemorrhoids directly correlates with number of pregnancies and deliveries (21, 28); 70% of women diagnosed with hemorrhoids had at least one previous pregnancy (18). It was determined that after the first pregnancy, hemorrhoids occur in 37.9% percent of women, and after other pregnancies this number increases (after two pregnancies, 38.4%, after three or more pregnancies, 40%) (28). In addition, hemorrhoids occur in 85% of nonprimiparous women (29, 30). Childbirth increases the risk of hemorrhoids almost eight times (31). There is an ongoing discussion regarding the method of delivery and pelvic floor dysfunction. Some studies suggest that women who experience vaginal delivery have a higher risk of developing pelvic floor dysfunction than women who undergo cesarean section, while others failed to demonstrate any benefit with cesarean section (32).

The method of delivery can cause hemorrhoids- women who give birth naturally (normal delivery) and in whom instrumental delivery is used are more likely to develop hemorrhoids as compared to women that undergo cesarean section. A study in which a three-dimensional perineal ultrasound scanning of the anal sphincter complex was performed found out that the delivery method has a certain influence on the shape of the anal sphincter complex. The thickness of the internal and external anal sphincter of primiparous women in a certain direction is significantly smaller than that by caesarean section (33). However, patients with a cesarean section history should be encouraged to give vaginal birth. Although the second stage of labor is usually extended but the incidence of third- and fourth-degree perineal lacerations is not increased (34).

Other risk factors related to the previous deliveries are prolonged birth (more than 12 hrs), prolonged second stage of labor (35, 36) and straining duration (4, 37), high weight of the newborn (4,000 g and more), spontaneous childbirth (38), and prolonged pregnancy (more than 40 weeks of pregnancy) (4, 37, 39).

The risk between constipation and hemorrhoids is established. Constipation during pregnancy definitely increases the risk of development of perianal diseases during pregnancy and up to sixfold after childbirth (31, 36). Upto 40% of women experience constipation during pregnancy (40–42). The risk of constipation is associated with the number of births—it is more common in nonprimiparous women (14). Risk factors of constipation during pregnancy can be divided into four groups: (1) dietary changes (iron supplements' consumption, insufficient fluid levels in the body due to nausea, and vomiting during pregnancy); (2) behavioral changes (decreased physical activity, physical, and social stress); (3) humoral changes that affect slower bowel movements (increased levels of progesterone and estrogen, decreased motilin concentration); (4) other causes (growing uterus due to pregnancy, painful hemorrhoids) (3). Almost every woman's nutrition changes during pregnancy. It is very important to maintain the intake of fluids, which is often insufficient, due to nausea and vomiting especially during the first trimester of pregnancy. Pregnant women less commonly use fiber-containing foods (21). The risk of constipation may also increase due to medications: iron preparations are used to treat anemia, under hypertensive conditions—magnesium sulfate is also commonly used (21). Increased body mass index (BMI) was described as a risk factor for hemorrhoids and perianal diseases during pregnancy and postpartum period (42, 43). Hemorrhoids are more common in the older pregnant women and mothers (21, 28). Symptoms of hemorrhoids and other perianal diseases progress during pregnancy, therefore many women experience reduced quality of life, especially in the third trimester of pregnancy and after childbirth (22). Constipation and hemorrhoids have strong negative effect both on the physical and emotional well-being of women's health and deteriorate their quality of life after childbirth (44).

## Perineal Trauma

Perineal trauma is a very common complication of vaginal delivery and plays an important role in pelvic floor dysfunction. Traumatic delivery appears to be associated with thrombosed external hemorrhoids (37).

Lacerations can occur spontaneously or iatrogenically, as with an episiotomy, on the perineum. Severe lacerations are associated with a higher incidence of long-term pelvic floor dysfunction, pain, dyspareunia, and embarrassment (45–47). Perineal lacerations are classified into four basic categories (47). First and second degree describes lacerations which involve the vaginal mucosa and perineal skin or body. Though those lacerations are quite superficial, women having second-degree lacerations are not at increased risk for pelvic floor dysfunction other than increased pain, and slightly lower sexual function scores at 6 months postpartum (48). Third degree is a second-degree laceration with the involvement of the anal sphincter. Fourth degree perineal laceration is described as third-degree laceration involving the rectal mucosa. Severe perineal lacerations, which include third- and fourth-degree lacerations, are referred to as obstetric anal sphincter injuries (OASI) (47). The most often used methods to decrease risk of perineal trauma are episiotomy and hands-on approach and perineal support. There is an ongoing debate regarding the routine vs. restrictive use of episiotomy. Both the World Health Organization and the American College of Obstetrics and Gynecologists recommended restricted use of episiotomy (47). Meanwhile some studies suggest that episiotomy can significantly reduce the number of genital lacerations and selective use of episiotomy is clinically feasible and effective (49, 50). This policy seems to be associated with a lower delivery-related perineal trauma as showed by the sub-classification, which could be a useful tool to monitor obstetric care (50). Moreover, hand on the fetal head and perineal support both were protective factors for OASI (51). Rising rates of obstetric anal sphincter injury (OASI) led to a collaborative effort by the Royal College of Obstetricians and Gynaecologists (RCOG) and the Royal College of Midwives (RCM) to develop and evaluate the OASI Care Bundle (OASI-CB). The OASI-CB comprises four practices (antenatal discussion about OASI, manual perineal protection, mediolateral episiotomy at 60° from the midline, and systematic examination of the perineum, vagina and ano-rectum after vaginal birth) and was initially implemented as part of a quality improvement (QI) project—“OASI1”—in 16 maternity units across Great Britain. Evaluation of the OASI1 project found that the care bundle reduced OASI rates and identified several barriers and enablers to implementation (52). Those methods used to prevent perineal trauma can help avoid traumatic delivery and reduce the risk of hemorrhoids after childbirth.

## Frequency

Prevalence and risk factors of anal diseases during pregnancy and after delivery were previously studied (53, 54). The most common perianal diseases during pregnancy and after childbirth are hemorrhoids and peri-anal fissure with frequency of 43.9% and the most common time of occurrence being the third trimester of pregnancy (61%) and the first or the second day after delivery (34 %) (1). A study of 280 women found that 114 (92.7%) of them had hemorrhoids, 7 (5.7%) of women had hemorrhoids and anal fissure, and 2 (1.6%) of women had anal fissure. Of the 121 studied women diagnosed with hemorrhoids, hemorrhoidal thrombosis was diagnosed for 64 (52.9%) women.

## Symptoms

The most common clinical symptoms of perianal diseases were pain, discomfort, itching, nodules, burning, mucus in the anus, and bleeding from anus. Hemorrhoids in pregnant women, as mentioned, can occur under two acute conditions:

1. Thrombosed Internal Hemorrhoids-Urgent Clinical Condition Accompanied by Intense Pain, When Nodules of Internal Hemorrhoids Suddenly get Stuck in the Anal Canal, Thrombosis of the Blood Vessels Occurs and Hemorrhoids Cannot be Reduced Back Into the Anal Canal for Several Days.
2. Perianal Venous Thrombosis-Subcutaneous Venous Thrombosis can be Painful; However the Main Symptom Is a Nodule Composed of a Clot Occurring in the Subcutaneous Tissue, Sometimes the Clot Stretches the Skin and Causes Necrosis and Perforation, With Subsequent Evacuation of the Clot and Spontaneous Recovery. In Some Cases, the Clot Gradually Disappears, Often Leaving Excessive Skin in the Anal Area.

## Treatment

In the absence of acute conditions, hemorrhoids like most other surgical diseases, are not treated before the end of the period of lactation.

Currently in Europe and the US, similar hemorrhoids' treatment guidelines are used (55). In all the cases of hemorrhoids, it is recommended to start with conservative treatment, with regulation of defecation. It is recommended to avoid constipation, long straining during defecation, and long sitting on the toilet. It is recommended to wash-up each time after defecation. Effective conservative treatment methods are flavonoids and topical preparations. In cases when a conservative treatment is ineffective, minimally invasive procedures can be tried: rubber band ligation, sclerotherapy, or infrared photocoagulation. Quite popular but more expensive dearterialization or stapled hemorrhoidopexy are not better than more traditional rubber band ligation and excisional hemorrhoidectomy interventions. In case of grade III-IV hemorrhoids with/or large external skin tags, surgical treatment is recommended (55). The search for new treatment methods of hemorrhoids is in continuation—laser hemorrhoidoplasty appears to be more effective than the suture hemorrhoidopexy but less effective than excision (56).

Most common acute perianal condition during pregnancy and after childbirth is perianal thrombosis and thrombosed internal hemorrhoids. Both diseases are characterized by a severe, sudden onset of pain forcing the seeking of medical help quickly. It is most commonly recommended to treat patients conservatively by prescribing adequate pain relief, oral, and topical flavonoid preparations. Warm sitz baths are recommended, which improve blood circulation in the anal tissue and reduce pain by reducing the internal anal sphincter tonus. Although most pregnant women experience resolution of their symptoms with the conservative methods mentioned above, some women will need surgical treatment.

In cases of large symptomatic perianal thrombosis, thrombectomy may be performed, ideally under local anesthesia (57). Also, surgical interventions in the presence of internal hemorrhoid thrombosis are not recommended, because of increased anal sphincter damage, and the increased risk of anal stenosis.

## Conclusions

Hemorrhoids and anal fissures occur in about 40% of pregnant women and women after delivery, usually in the third trimester of pregnancy and 1–2 days after giving birth. Constipation during pregnancy, perianal diseases during previous pregnancy and childbirth, instrumental delivery, straining duration of more than 20 min, and newborn weight of more than 3,800 g are associated with hemorrhoids. Perianal diseases reduce the quality of life of women. In the absence of acute conditions, surgical treatment of hemorrhoids is delayed after pregnancy, childbirth, and lactation. Thrombosed internal hemorrhoids and perianal thrombosis are to be treated conservatively in most instances by prescribing adequate pain relief, oral, and topical flavonoid preparations.

## Author Contributions

DB is the designated first author and TP is the designated senior author of the publication. All authors drafted parts of the article and reviewed the article and agree with the final version of the manuscript.

## Conflict of Interest

The authors declare that the research was conducted in the absence of any commercial or financial relationships that could be construed as a potential conflict of interest.

## Publisher's Note

All claims expressed in this article are solely those of the authors and do not necessarily represent those of their affiliated organizations, or those of the publisher, the editors and the reviewers. Any product that may be evaluated in this article, or claim that may be made by its manufacturer, is not guaranteed or endorsed by the publisher.
